# Improvements in the Bureau of Animal Industry

**Published:** 1900-09

**Authors:** 


### IMPROVEMENTS IN THE BUREAU OF ANIMAL INDUSTRY.

The service of the Bureau of Animal Industry is constantly being made more desirable for veterinarians. Beginning with this year, a leave of absence of two weeks with pay is allowed each year.

Veterinarians secure appointment at $1200 per annum almost as soon as they have passed the civil-service examination. Those who passed the regular examination in April were all appointed within a few weeks of the time their papers were passed upon. A special examination was held in July, and all who passed then have received appointments. The regular fall examination will be held at various points throughout the country on October 23d, and persons desiring to take the same should make application to the Civil-Service Commission at Washington at least two or three weeks in advance of that date.

There are now in the Bureau service 275 veterinarians, and further appointments will be made as soon as the next civil-service examination has been held.

The following promotions have been made since January 1, 1899:

| | | | | |
|---|---|---|---|---|
| Dr. Louis Abel, New York City, | from | $1200 | to | $1400 |
| Dr. Harry B. Adair, Kansas City, | " | " | | " |
| Dr. Leslie J. Allen, Kansas City, | " | " | | " |
| Dr. O. G. Atherton, Chicago, | " | " | | " |
| Dr. Lewis R. Baker, Hammond, Ind., | " | 1400 | to | 1600 |
| Dr. S. E. Bennett, Kansas City, | " | 1600 | to | 1800 |
| Dr. Thomas A. Bray, El Paso, Texas | " | 1200 | to | 1400 |
| Dr. Lowell Clarke, South Omaha, | " | " | | " |
| Dr. Louis P. Cook, Cincinnati, | " | " | | " |
| Dr. S. E. Cosford, Lincoln, Neb., | " | " | | " |
| Dr. E. T. Davison, Leavenworth, Kansas, | " | " | | " |
| Dr. L. Enos Day, Waterloo, Iowa, | " | " | | " |
| Dr. F. L. DeWolf, Chicago, | " | " | | " |
| Dr. George Ditewig, Davenport, Iowa, | " | " | | " |
| Dr. O. E. Dyson, Chicago, | " | 1600 | to | 1800 |
| Dr. A. T. Everett, South Omaha, | " | 1200 | to | 1400 |
| Dr. George C. Faville, Norfolk, Va., | " | 1400 | to | 1600 |
| Dr. Nathan K. Fegley, Jersey City, | " | 1200 | to | 1400 |
| Dr. James Fleming, Chicago, | " | " | | " |
| Dr. Lewis K. Green, Detroit, | " | " | | " |
| Dr. H. W. Hawley, Chicago, | " | " | | " |
| Dr. F. W. Hopkins, Cairo, Ill., | " | " | | " |
| Dr. U. G. Houck, Chicago, | " | " | | " |
| Dr. E. N. Hutchinson, Portland, Ore., | " | " | | " |
| Dr. G. A. Johnson, Sioux City, Iowa, | " | " | | " |

| | |
|---|---|
| Dr. James Johnston, Boston, | from $1200 to $1400 |
| Dr. Benj. F. Kaupp, Kansas City, | " " " |
| Dr. Charles Keane, Los Angeles, Cal., | " " " |
| Dr. James S. Kelly, Quincy, Ill., | " " " |
| Dr. F. D. Ketchum, South St. Paul, | " " " |
| Dr. R. R. Letts, New York City, | " " " |
| Dr. A. McBride, Jersey City, | " " " |
| Dr. Harry G. Moore, South St. Joseph, | " " " |
| Dr. W. J. Murphy, Brightwood, Mass., | " " " |
| Dr. Don W. Patton, South Omaha, | " " " |
| Dr. A. G. G. Richardson, Boston, | " 1600 to 2000 |
| Dr. O. W. Snyder, Eau Claire, Wis., | " 1200 to 1400 |
| Dr. R. P. Steddom, Washington, | " " " |
| Dr. Walter J. Stewart, Milwaukee, | " " " |
| Dr. E. L. Volgenau, New Haven, | " " " |
| Dr. J. Wilson, South Omaha, | " " " |
| Dr. Morris Wooden, Chicago, | " 1400 to 1800 |
| Dr. C. H. Zink, Buffalo, | " 1400 to 1600 |

